# Xylogenesis and phloemogenesis in the flesh of sweet cherry fruit are limited to early-stage development

**DOI:** 10.1038/s41598-022-16544-1

**Published:** 2022-07-18

**Authors:** Jonas Gärtner, Eckhard Grimm, Moritz Knoche

**Affiliations:** Institute for Horticultural Production Systems, Leibniz-University Hannover, Herrenhäuser Straße 2, 30419 Hannover, Germany

**Keywords:** Plant development, Developmental biology, Plant sciences, Structural biology

## Abstract

Water inflows into sweet cherry fruit occur via the xylem and the phloem vasculatures of the pedicel. The rates of these inflows are subject to marked changes during fruit development. The objective was to establish if, and when, xylogenesis and phloemogenesis occur in the fruit flesh (mesocarp) during fruit development. Fruit were cut in half and the median and the lateral bundles inspected by light microscopy. Fruit mass increased with time in a double sigmoid pattern. Xylogenesis and phloemogenesis were both limited to early fruit development (stage I). There were no consistent changes in the areas of either xylem or phloem after stage I until maturity (i.e., during stages II and III). The cross-sectional areas of xylem and of phloem in a bundle were both linearly related to total bundle area. Most of the increases (stage I) in bundle area (62%, r^2^ = 0.99***) were accounted for by increases in phloem area and about 35% (r^2^ = 0.97***) by increases in xylem area. A small percentage of the xylem area increase (about 4% of the total area of the bundle; r^2^ = 0.48***) was contributed by the appearance of intercellular spaces within the xylem. Our results suggest, that new xylem and phloem tissues are differentiated only during early development.

## Introduction

Fruit water balance is key to the growth and development of any fruit, including that of sweet cherry. There are a number of pathways for water to enter and to leave a sweet cherry fruit, with fruit water content continuously integrating all these incoming and outgoing water fluxes. The main fluxes are those due to the (convectional) xylem and phloem sap flows between the tree and the fruit via the fruit pedicel and the (diffusional) transpiration and osmotic exchanges between the fruit and the atmosphere via the fruit skin. The xylem and phloem sap flows are mostly in the direction from tree to fruit, but the xylem sap does sometimes flow in the opposite direction – i.e., from fruit to tree^1^. Meanwhile, water exits the fruit via its skin due to transpiration (mostly but not exclusively during the daytime when the vapour pressure deficit of the atmosphere is greatest) and it enters the fruit by osmosis (but only during periods when the fruit skin is wet with rain or dew). These osmotic flows are very important in relation to fruit skin integrity (i.e., to cuticular microcracking and skin rain-cracking^2^) but, with the exception of rains, they are small in relation to the fruit water balance^3^. But this study focuses just on the xylem and phloem sap flows.

Recent investigations^1^ have established that water inflow via the pedicel is subject to marked changes during the course of development. During early fruit development (stage I), volume growth of stone fruit species, such as sweet cherry, is relatively slow and due primarily to cell division in the pericarp^4,5^. During this stage I period, the water inflow required to support the gradual increase in fruit volume and also to compensate for the fruit’s transpirational water losses, is substantially due to the water entering the fruit in the xylem sap. During this stage of growth the contribution to total water inflow made by the phloem is relatively small. However, as fruit development proceeds into the next phase (stage II), the pit develops, with very little change in fruit mass (volume). Water inflow via the xylem now begins to decrease, whereas water inflow via the phloem begins to increase. During the subsequent phase of growth (stage III, also termed final swell), fruit volume increases rapidly due to cell expansion primarily in the mesocarp. Stage III development is characterised by a colour change from green, through yellow, to red/black) and by a rapid decrease in the osmotic potential (more negative) of the fruit’s expressed juice, as a result of the import of carbohydrates in the phloem sap. In this stage, the phloem becomes the dominant pathway for water inflow to the fruit. By maturity (end stage III), nearly all the water inflow to the fruit through the pedicel is via the phloem, with almost no water inflowing via the xylem^1,6^. Similar observations were reported for apples^7^.

The reason for the marked decrease in xylem inflow is due to physical rupture of the xylem vessels^6^. This leaves the phloem as the primary pathway for entry of water and carbohydrates to the fruit. The evidence for this change in function is based on (1) measurement of fruit diameter changes using linear variable displacement transducers and steam girdling of the pedicel (which kills the phloem)^1^, (2) potometry using detached fruit fed with stains such as methylene blue and acid fuchsin^6,8^, and (3) magnetic resonance imaging of detached fruit fed with gadoteric acid^6^.

While the changes in the functionality of the xylem and phloem vasculatures are well established, there is little information on the anatomical background. For example, it is not known whether the increase in phloem sap flow into developing fruit is due, fully or in part, to the differentiation of new sieve tubes (phloemogenesis) – or whether these are simply more active. Also, it is not known whether the differentiation of some new xylem vessels (xylogenesis), partly compensates for the rupture of the older xylem vessels.

This study was undertaken to identify the anatomical background for the observed changes in vascular flow rates via the pedicel, with respect to the state of the xylem and phloem tissues within the fruit. We focussed on the median and the two lateral bundles since these (1) supply the fleshy mesocarp and (2) progressively decline in functionality from the stage II/III transition onwards due to rupture^6^.

## Results and discussion

**Figure 1:**
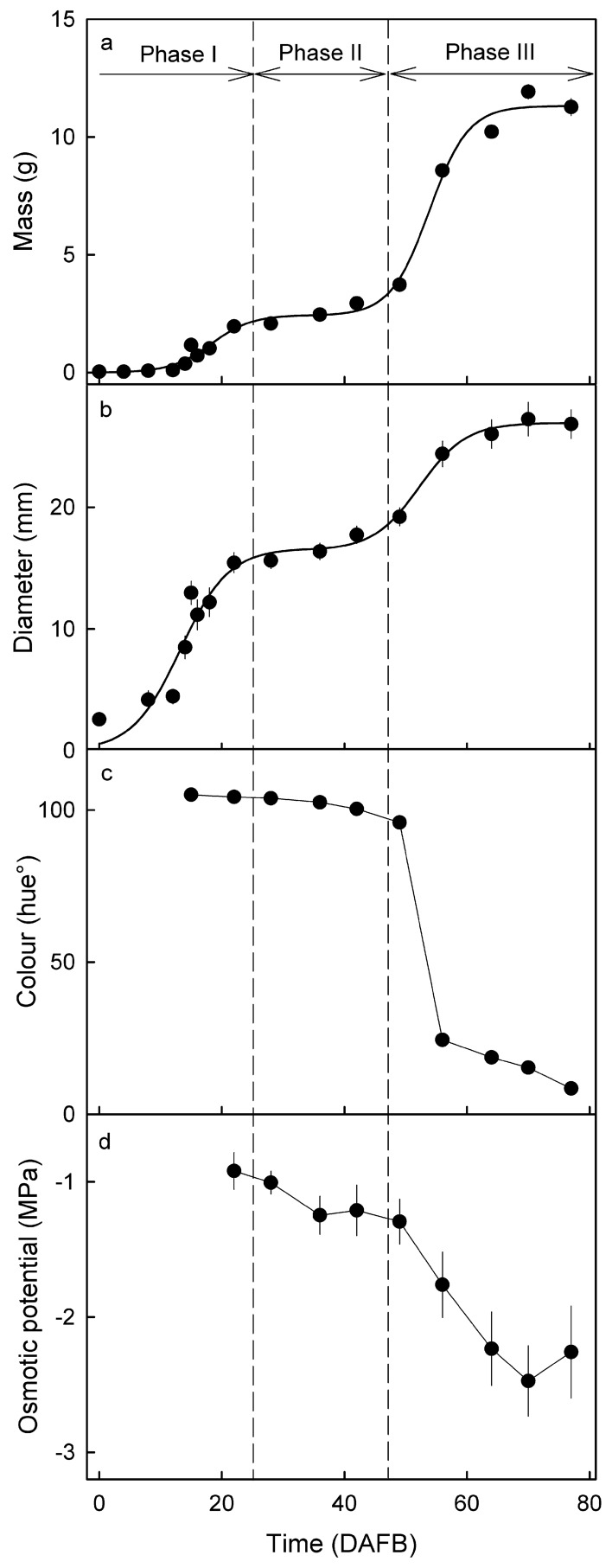
Developmental time course of change in mass (**a**), diameter (**b**), colour as indexed by the hue angle (**c**), and the osmotic potential of the expressed juice (**d**). The *x*-axis scale in days after full bloom (DAFB). Vertical dashed lines indicate stage I/II and stage II/III transition.

Fruit mass and mean fruit diameter increased with time, following a double sigmoid pattern (Fig. 1a, b). The colour change from green via yellow to red began at about 50 days after full bloom (DAFB) (Fig. 1c). The osmotic potential of the juice decreased slightly (more negative) from 22 to 49 DAFB, and more markedly from 49 to 80 DAFB (maturity) (Fig. 1d). Based on the growth pattern, time of colour change and the beginning of osmolyte accumulation, the stage I/II transition occurred at about 25 DAFB, the stage II/III transition at about 42 DAFB.

**Figure 2:**
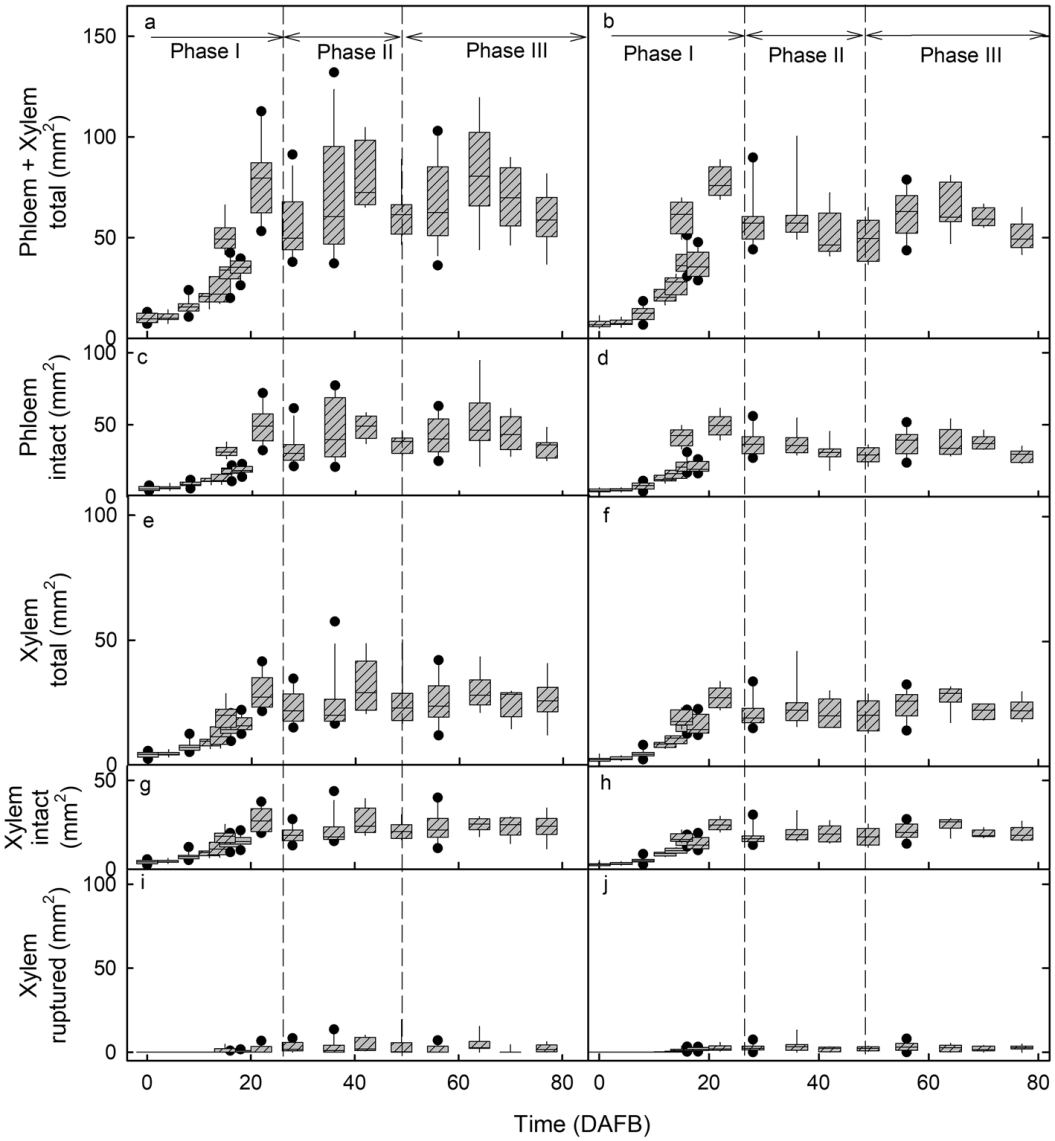
Developmental time course of change in cross-sectional areas of the median bundle (**a, c, e, g, i**) and the lateral bundle (**b, d, f, h, j**). (**a, b**) Whole bundle comprising xylem plus phloem. (**c, d**) Phloem. (**e, f**) Total xylem. (**g, h**) Intact xylem. (**i,j**). Ruptured xylem. The *x*-axis scale in days after full bloom (DAFB). The box represents the 25th and 75th percentiles, the horizontal line in the box the median, and the whiskers the 5th and 95th percentiles. Individual symbols beyond the whiskers are outliers.

As judged from change in the measured cross-sectional areas of whole vascular bundles (i.e., containing both xylem and phloem), both xylogenesis and phloemogenesis were limited to stage I of fruit development. The areas of the median and lateral bundles both increased only during stage I and remained constant during the subsequent stages II and III (Fig. 2a, b). Except for the greater variability in cross-sectional area of the median bundle, there were no consistent differences in bundle area between the median and lateral bundles (Fig. 2). These findings are consistent with the decrease in xylem functionality reported earlier ^1,6,8^. The decrease in xylem functionality under constant cross-sectional area was thus due to the progressive rupture of the vessels^6^. We detected no evidence of xylogenesis, nor of any repair of ruptured vessels.

The question arises as to why the phloem apparently remains functional and intact as indicated by its increasing contribution to total water inflow during development, whereas the xylem (immediately adjacent) ruptured through growth-induced straining. It is speculated that the xylem, a system of dead sclerenchyma tubes with thick encrusted cell walls, has a higher modulus of elasticity (stiffer) and a lower fracture strain, compared to the living cells of the phloem. Which we assume are more extensible with a lower modulus of elasticity (less stiff) and a higher fracture strain. The strain released following excision of tissue blocs of the inner flesh (containing the median bundle) averaged 24% in early stage III and 44% at maturity^6^. Unfortunately, our attempts to directly measure the strain relaxation properties of enzymatically isolated vascular bundles were not successful (Grimm, unpublished data).

The measured areas of the xylem and phloem tissues were a linear function of the measured areas of the whole bundle (which is comprised of these two tissues). This finding was consistent whether we analysed the median bundle or one of the two lateral bundles. Plotting the phloem area (intact) of the bundle against the total bundle area (phloem + xylem) revealed that most of the (stage I) bundle area increase (62%, r^2^ = 0.99***) was accounted for by an increase in phloem area. For the (intact) xylem area the contribution to total bundle area was only about 35% (r^2^ = 0.97***) (see Supplementary Fig. S1 online). A small percentage of xylem area (about 4% of the total area of the bundle; r^2^ = 0.48***) was contributed by the appearance of intercellular spaces (see Supplementary Fig. S1 online). It is speculated that the appearance of intercellular spaces in the xylem are due to vessel rupture and subsequent elastic axial contraction and so retreat.

**Figure 3:**
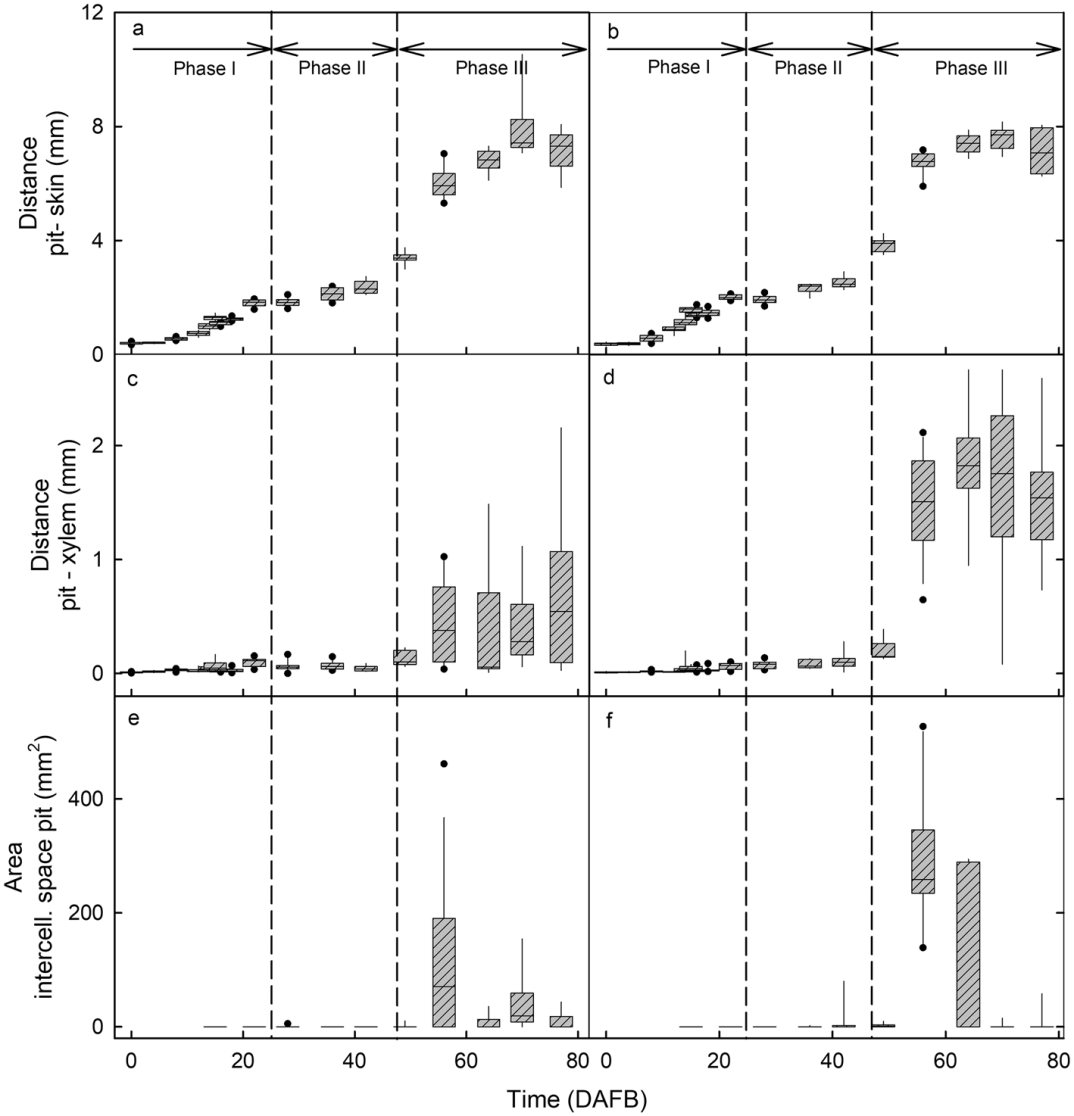
Developmental time course of changes in distances and cross-sectional areas of the median bundle in the cheek region (**a, c, e**) and the lateral bundles in the shoulder regions (**b, d, f**). (**a, b**) Distance between pit and skin. (**c, d**) Distance between pit and xylem. (**e, f**) Areas of the intercellular spaces around the pit. The *x*-axis scale in days after full bloom (DAFB). The box represents the 25th and 75th percentiles, the horizontal line in the box the median, and the whiskers the 5th and 95th percentiles. Individual symbols beyond the whiskers are outliers.

The change in distance between pit and skin with development mirrored the double sigmoidal growth pattern obtained for the change in fruit mass and fruit diameter (Fig. 3a,b). There was no difference in expansion between the cheek or the shoulder regions (Fig. 3a,b). Part of the increase in diameter was accounted for by an increase in the distance between the pit and the xylem of the vascular bundle (Fig. 3c,d).

**Figure 4:**
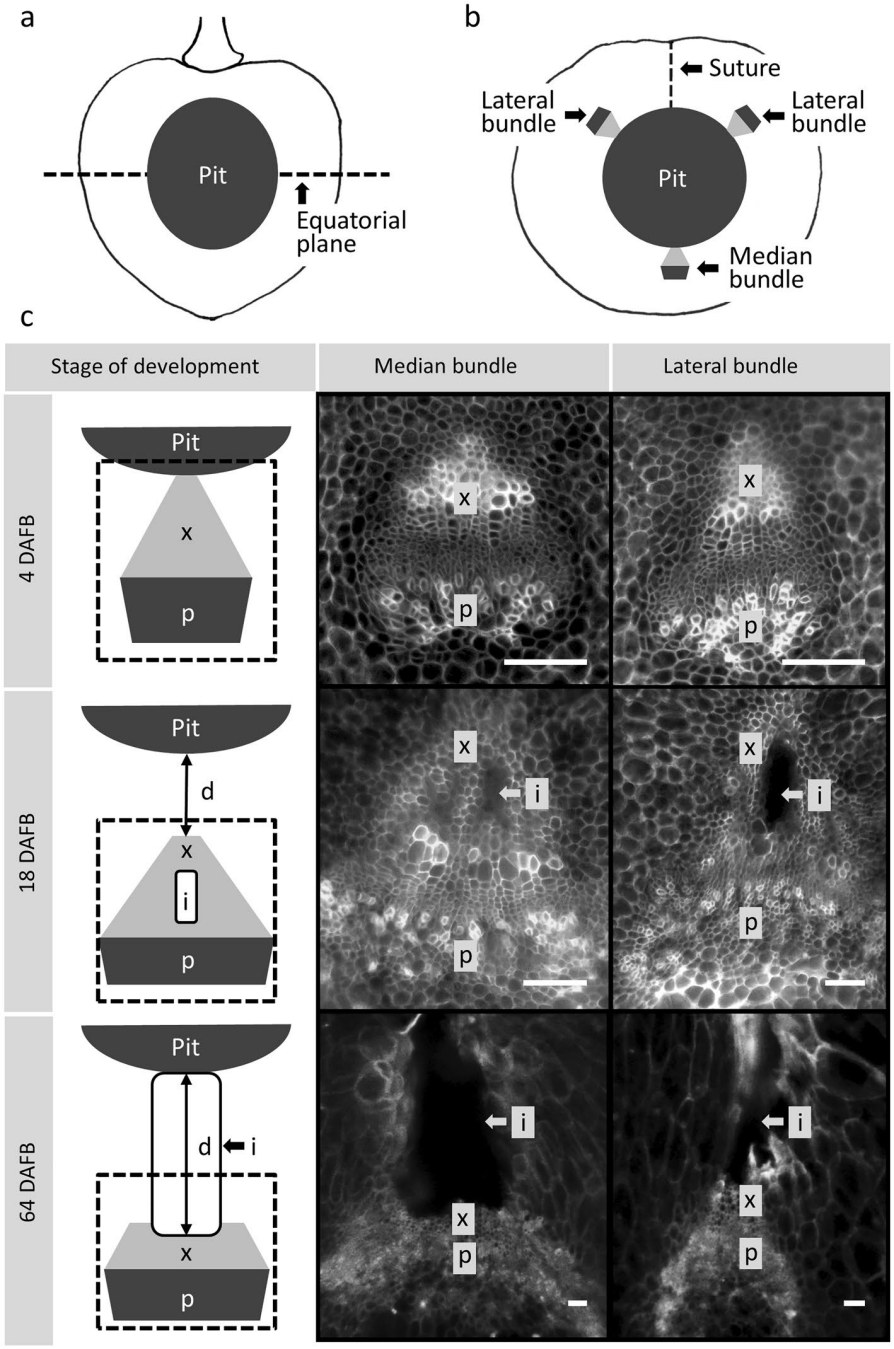
(**a, b**) Diagram of sweet cherry fruit. (**a**) Longitudinal section. (**b**) Cross-section in the equatorial plane indicating positions of the suture, the two lateral bundles and the median bundle. The dashed line in **a** indicates equatorial plane. (**c**) Sketch (left column) and corresponding micrographs of cross-sections through the median bundle (centre column) and lateral bundle (right column) at an early stage I (4 days after full bloom (DAFB), top row), an intermediate stage (18 DAFB, centre row), and a mature stage III (64 DAFB, bottom row). Scale bar 50 μm, i = intercellular space, p = phloem, x = xylem, d = distance pit – xylem, dashed boxes in diagrams indicate position of the microscope window.

Rupture of xylem vessels resulted in the formation of intravascular intercellular spaces in the xylem. These spaces were absent during early stage I (Fig. 2i,j; Fig. 4c 4 DAFB) but increased in size from stage II onwards (Fig. 2i,j; Fig. 4c 18 DAFB). In addition, during stage III, intercellular spaces also formed outside the vascular tissue between the bundles and the pit (Fig. 3e,f; Fig. 4c 64 DAFB). These extravascular spaces eventually merged with the intravascular ones. The onset of stage III marks a period of rapid increase in fruit volume growth, so an increased rate of strain for the bundles as they are embedded in an expanding flesh tissue. It is this strain that is likely responsible for the increase in intercellular air spaces surrounding the pit.

As the fruit continues to grow, the mesocarp becomes compressed (tissue pressure) by the strained fruit skin, resulting in decreases in the number and size of the intercellular spaces—some of which disappear in late stage III (Fig. 3e,f). This interpretation is consistent with the occurrence of marked skin strain during stage III as indexed by strain-relaxation analysis of skin samples following excision, by the ‘gaping’ of cherry fruit following incision^9^ and also by the occurrence of strain spots^10^. It is also consistent with the occurrence of ruptured vessels in the xylem during stage III development^6^. An increase in stress during stage III also provides an explanation for the separation of the median and lateral bundles from the pit and their lateral ‘migration’ that results in the formation of major intercellular spaces immediately adjacent to the pit. The schyzogenous response to increased strain, the separation from the pit and the axial failure of individual vessels would (as expected) occur somewhat randomly as indicated by the high variability in the numbers, distribution and sizes of the intercellular spaces (Fig. 3c-f). These findings are consistent with those reported for apple by Drazeta et al.^7^.

Our results confirm that: (1) the differentiation of new xylem and phloem during fruit development is limited to stage I; and (2) that there is no evidence for xylogenesis or phloemogenesis during stages II or III. Moreover, these observations are consistent with the marked decline in xylem functionality reported earlier. However, it would seem that the mechanical properties of the (living) phloem tissues are such that they are better able to cope with the growth strains associated with the rapid stage III growth of a sweet cherry fruit, than the (lignified, substantially dead) xylem tissues.

## Material and methods

### Plant material

‘Sam’ sweet cherry (*Prunus avium* L.) trees grafted on 'Gisela 5' rootstocks (*P. cerasus* L. × *P. canescens* Bois) were cultivated under a rain shelter at the Horticultural Research Station of the Leibniz University Hannover in Ruthe (lat. 52°14′N, long. 9°49′E) according to current regulations for integrated crop production. Trees were trained to a central leader, the planting distance was 2.5 m × 5.5 m. Cherry fruits were sampled randomly from a total of 41 trees between 0 and 77 DAFB. Only fruit of representative size and colour and without visual defects were selected. Fruit were harvested in the mornings, held under non-transpiring conditions and carried to the laboratory.

### Fruit development

The pedicel was cut to a standard length of 2–3 mm. Skin colour was determined in the L*a*b* colour space using a spectrometer (CM-2600d; Konica Minolta, Tokyo, Japan). The Hue angle was calculated. Fruit fresh weight and the three orthogonal diameters were recorded. Mean fruit diameter was calculated as the geometrical mean of these three measures. Juice was extracted from a representative subsample of fruit using a garlic press and its osmolarity quantified by water vapor pressure osmometry (VAPRO® 5600, Wescor, Logan, UT). All measurements were carried out with a minimum number of 19 single-fruit replicates, only osmolarity was measured with 10 replicates. Whole fruit were incubated in Karnovsky fixative and held at 2 °C until further processing^11^.

### Microscopy

Fruit were cut in half in the equatorial plane using a sharp razor blade to expose a smooth flat tissue surface. Until 22 DAFB the pit was soft, so enabling this. From 22 DAFB onwards the pit began to harden and had to be removed from the sectioned halves. The half fruits were surface stained for 1 min using 0.5% w/v calcofluorwhite (this stains the cell walls, and particularly, the thickened walls of the xylem vessels). Following staining, samples were rinsed with deionized water, covered with a coverslip and viewed under a light microscope (MZ10F; Leica Microsystems GmbH, Wetzlar, Germany; BX-60; Olympus, Hamburg, Germany) under incident bright light and incident UV light. Calibrated micrographs of the median and the lateral bundles were taken (DP73; Olympus, Germany) (Fig. 4a). Sweet cherry fruit have five major axial bundles. Of these the median one runs on the cheek opposite of the suture, the two lateral ones run on opposite sides of the shoulders from the pedicel towards the stylar scar^6^. The two remaining bundles connect to the pit. The number of fruit inspected at anyone sampling time ranged from 8 to 14 for the median bundle and from 7 to 12 for the two lateral bundles. Data for the lateral bundles of each fruit were averaged.

### Image analysis

The micrographs were analysed using image analysis (cellSens Dimension 1.7.1; Olympus). The areas of a whole vascular bundle and its phloem and xylem components and any intercellular spaces associated with the xylem were quantified by manually tracing the respective perimeters. The xylem tissue was identified by its location, facing the pit, by the blue fluorescence of the large vessels and by their thick cell walls. The phloem tissue was identified by its location adjacent to the xylem, facing the skin, and by its small cells. Intercellular spaces were identified as bright, cell-free empty voids in the tissue (for sketch see Fig. 4). A lack of intercellular spaces is interpreted as indicative of an intact phloem or xylem tissue at the respective stage of development (in general both the xylem and the phloem tissues are without obvious airspaces). Intercellular spaces were located within the xylem, or adjacent to the xylem between xylem and pit. The latter are referred to as ‘intercellular spaces around the pit’. The distances between the outside of the pit and the skin, and between the outside of the pit and the innermost part of the xylem were measured (for diagram see Fig. 4c).

### Data analyses

All experiments were performed in accordance with relevant guidelines and regulations. The data points presented in the figures are shown as means ± standard errors (Fig. 1), as boxplots (Figs. 2,3). In the boxplots, the box represents the 25^th^ and 75^th^ percentiles, the horizontal line in the box the median, and the whiskers the 5^th^ and 95^th^ percentiles. Individual data symbols beyond the whiskers are outliers. Analyses of variance and regression were carried out using the statistical software package SAS (version 9.1.3; SAS Institute Inc, Cary, N.C.). Means were compared using Tukey’s multiple range test. Significance of coefficients of correlation (r) and of determination (r^2^) at the 5, 1 and 0.1% levels are indicated by *, ** and ***, respectively.

## Supplementary Information

Supplementary Information 1.

Supplementary Information 2.

## Data Availability

All data generated or analysed during this study are included in this published article and its supplementary excel file.
