# Supplementary material for: Xylogenesis and phloemogenesis in the flesh of sweet cherry fruit are limited to early-stage development
Source: Sci Rep. 2022 Jul 18;12:12274. doi: 10.1038/s41598-022-16544-1 (PMC9293894; doi:10.1038/s41598-022-16544-1)
Supplement: Supplementary file 1 — Supplementary Information 1. [file 41598_2022_16544_MOESM1_ESM.docx]

**Supplementary Figure S1.** Relationship between the area of intact phloem (**a**), intact xylem (**b**) and ruptured xylem (**c**) and the total area of xylem plus phloem. The slope of the regression lines multiplied by 100 gives the percentage of the total bundle area accounted for by intact phloem, intact xylem and ruptured xylem. For details see text.
